# Supplementary material for: Integrative Gene Expression and Metabolic Analysis Tool IgemRNA
Source: Biomolecules. 2022 Apr 16;12(4):586. doi: 10.3390/biom12040586 (PMC9029533; doi:10.3390/biom12040586)
Supplement: Supplementary file 1 [file biomolecules-12-00586-s001.zip › biomolecules-1663931-supplementary file S1.pdf]

Supplementary file S1

Supplementary file S1

Classifying previously published transcriptome data integration in GSM methods

Table S1. Comparison of methods for transcription data integration

Name	Year	Returns a context - specific model	Thresholding	Gene mapping	Data Requirements	Is maintained nowadays	Availability in third party software
Akesson	2003	No	Yes, one user-specified threshold	Not found	A genome-scale metabolic network reconstruction.	No	Not found
Gene Inactivity Moderated by Metabolism and Expression (GIMME)	2008	Yes	Yes, one user-specified threshold	Expression data has to be mapped to reactions before calling the algorithm, can be done using Cobra Toolbox pre-processing functions	1. A genome-scale metabolic network reconstruction,<br>2. specified objective function,<br>3. one threshold,<br>4. one gene expression data set,<br>5. calculated reaction expression levels (gene mapping)	Yes	MATLAB, Cobra Toolbox
Integrative metabolic analysis tool (iMAT)	2008	Yes	Yes, two user-specified thresholds (lower threshold and upper threshold)	Expression data has to be mapped to reactions before calling the algorithm, can be done using Cobra Toolbox pre-processing functions	1. A genome-scale metabolic network reconstruction,<br>2. two specified thresholds,<br>3. one expression data set,<br>4. calculated reaction expression levels (gene mapping)	Yes	MATLAB, Cobra Toolbox
E-Flux	2009	Flux distribution only	Yes, one user-specified threshold	Not found	1. A genome-scale metabolic network reconstruction,	Not found	Not found

Supplementary file S1

					2. a function to convert expression levels into an upper bound on fluxes,<br>3. gene expression data,<br>4. threshold		
Probabilistic regulation of metabolism (PROM)	2010	Flux distribution only	Yes,<br>one predefined low threshold (default: 33rd percentile)	Not found	1. A genome-scale metabolic network reconstruction,<br>2. a range of expression data from various environmental and genetic perturbations,<br>5. transcriptional regulatory network	Not found	Not found
Toolbox for Integrating Genome-scale Metabolism, Expression, and Regulation (TIGER)	2011	Yes	Facilitated GIMME, iMAT and MADE methods	Yes,<br>converting GPR associations into mixed integer linear program (MILP)	1. A genome-scale metabolic network reconstruction,<br>2. gene expression data,<br>3. transcriptional regulatory network	Not found	Not found
Metabolic Adjustment by Differential Expression (MADE)	2011	Flux distribution only	No	Not found	4. A genome-scale metabolic network reconstruction,<br>5. two or more gene expression data sets<br>3. a mixed-integer linear program solver	No	Linux / Windows/ MacOsX<br>MATLAB (requires a mixed-integer linear program solver)<br><br>(The most recent version of MADE, along with other tools for integrating expression data, is available as part

Supplementary file S1

							of the TIGER software package.)
Integrative Network Inference for Tissues algorithm (INIT)	2012	Yes	Yes, optional minimum flux threshold for expressed reactions (default 1e-8) and positive or negative weights for each reaction	Expression data has to be mapped to reactions before calling the algorithm, can be done using Cobra Toolbox pre-processing functions	1. A genome-scale metabolic network reconstruction,<br>2. gene expression data	Yes	MATLAB, Cobra Toolbox
Lee–12	2012	Flux distribution only	Not found	Not found	1. A genome-scale metabolic network reconstruction,<br>3. gene expression data	Not found	Cobra Toolbox (MATLAB)
Fang–12	2012	Flux distribution only	Not found	Not found	Not found	Not found	Not found
RELATive CHange (RELATCH)	2012	Flux distribution only	Not found	Not found	2. Gene expression data,<br>3. fluxomic data from a reference state	Not found	Not found
Temporal Expression-based Analysis of Metabolism (TEAM)	2012	Flux distribution only	Yes, threshold determination using background M3D data set	Not found	1. Initial media composition data,<br>2. temporal gene expression data<br>1. temporal biomass data	Accessible but only for microarray experiment s	Not found
Gene-expression FBA (GX–FBA)	2012	Flux distribution only	Not found	Not found	3. Not found	Not found	Not found
metabolic Context-specificity Assessed by Deterministic	2012	Yes	Yes, uses a core set of reactions that should be active based on gene	Mapped protein staining data to reactions according to gene-reaction associations and set a reaction score	1. A genome-scale metabolic network reconstruction,<br>2. gene expression data	Yes, code is open source	MATLAB, Cobra Toolbox

Supplementary file S1

Reaction Evaluation (mCADRE)			expression evidence and a sensitivity parameter: ratio cutoff				
Transcriptional regulated flux balance analysis (TRFBA)	2016	Limits upper bounds of reactions and predicts growth rate	No	Yes	1. A genome-scale metabolic network reconstruction,<br>2. set of expression data for various perturbations,<br>3. transcriptional regulatory network	Yes, code is open source	MATLAB, Cobra Toolbox
ΔFBA (deltaFBA)	2021	Yes	Yes, one global threshold	Using AND/OR = MIN/MAX GPR rule	1. A genome-scale metabolic network reconstruction,<br>2. gene expression data from two different conditions,<br>3. threshold	Yes	MATLAB, Cobra Toolbox
IgemRNA	2021	Yes	Yes, possible thresholds:<br>1. Global T1 (GT1),<br>2. Local T1 (LT1),<br>3. Local T2 (LT2)	Included, possible options:<br>1. Only irreversible reactions,<br>2. All reactions,<br>3. Growth not affecting gene deletion only,<br>4. Meet minimum growth requirements,<br>5. AND/OR = MIN/MAX,<br>6. AND/OR = MIN/SUM,<br>7. AND/OR = GM/MAX,<br>8. AND/OR = GM/SUM	3. A genome-scale metabolic network reconstruction,<br>4. gene expression data,<br>5. external metabolite uptake data (optional),<br>6. threshold/-s	Yes	MATLAB, Cobra Toolbox
