# Supplementary material for: Integrative Gene Expression and Metabolic Analysis Tool IgemRNA
Source: Biomolecules. 2022 Apr 16;12(4):586. doi: 10.3390/biom12040586 (PMC9029533; doi:10.3390/biom12040586)
Supplement: Supplementary file 1 [file biomolecules-12-00586-s001.zip › biomolecules-1663931-supplementary file S2.pdf]

Supplementary file S2

Supplementary file S2

User manual documentation for the IgemRNA

IgemRNA is a library with a graphical user interface written for the MATLAB environment and facilitates some of the Cobra Toolbox 3.0 functionality. *IgemRNA* performs not only Gene sets enrichment analysis-based functions, but also allows integrate transcriptomics data in metabolic models. Also, *IgemRNA* allows validate transcriptomics data facilitating interconnectivity of biochemical networks, steady state assumptions, Gene - Protein - Reaction relationship and can use optional medium composition data to create context-specific models.

1. Folder structure description

Files are extracted from the archive (https://github.com/BigDataInSilicoBiologyGroup/IgemRNA). The *IgemRNA* tool consists of four root folders (*Data, Scripts and Results non-optimization, Results post-optimization*) and an *IgemRNA.m* file which calls the user graphical interface form. Data folder is where the input data files are stored, initially this folder contains the data files used for this demonstration:

- MediumData.xlsx (medium composition data);
- Yeast_8_4_0.xls (the yeast consensus genome-scale model)
- TranscriptomicsData.xlsx (RNA-req measurements) (available https://www.ncbi.nlm.nih.gov/geo/query/acc.cgi?acc=GSE130549)

Transcriptomics data and medium composition data can be provided as an .xls or an .xlsx file and must meet the following format (Figure S1) where shown columns are provided and named accordingly and sheet names correspond to a phenotype name see 3.2 *IgemRNA* demonstration.

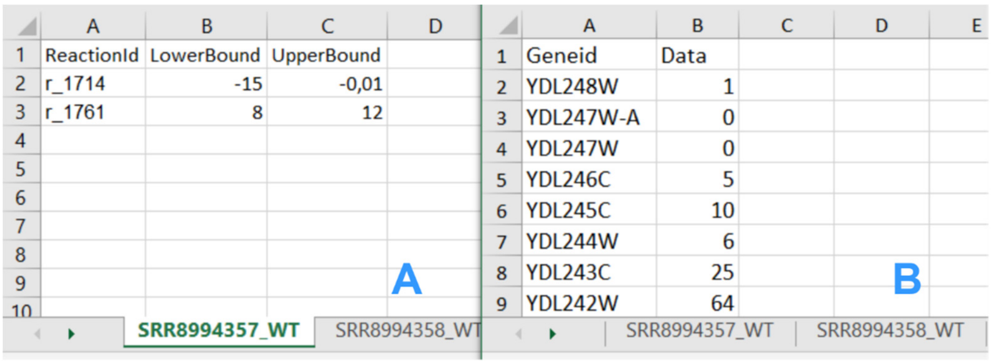

A

	A	B	C	D
1	ReactionId	LowerBound	UpperBound	
2	r_1714	-15	-0,01	
3	r_1761	8	12	

Sheets: SRR8994357_WT, SRR8994358_WT

B

	A	B	C	D	E
1	Geneid	Data			
2	YDL248W	1			
3	YDL247W-A	0			
4	YDL247W	0			
5	YDL246C	5			
6	YDL245C	10			
7	YDL244W	6			
8	YDL243C	25			
9	YDL242W	64			

Sheets: SRR8994357_WT, SRR8994358_WT

Figure S1. Input data file structure; (A) Medium data file structure; (B) Transcriptomics data file structure.

The model can be provided in xls, sbml or other formats supported by Cobra Toolbox 3.0.

Scripts folder consists of all the script files that are being executed by the *IgemRNA* tool according to user's selections in the *IgemRNA* form as well as the test cases provided in this demonstration. The Results non-optimization and Results post-optimization folders are where all the result files are being saved. These folders are initially empty (for more details see section in main publication Materials and Methods 2.2 Tools functionality description).

Supplementary file S2

2. Starting *IgemRNA* tool

In order to start the *IgemRNA* tool a user must start the MATLAB environment and run the *IgemRNA.m* script located in the root folder. This script opens the graphical user interface of *IgemRNA* (Figure S2).

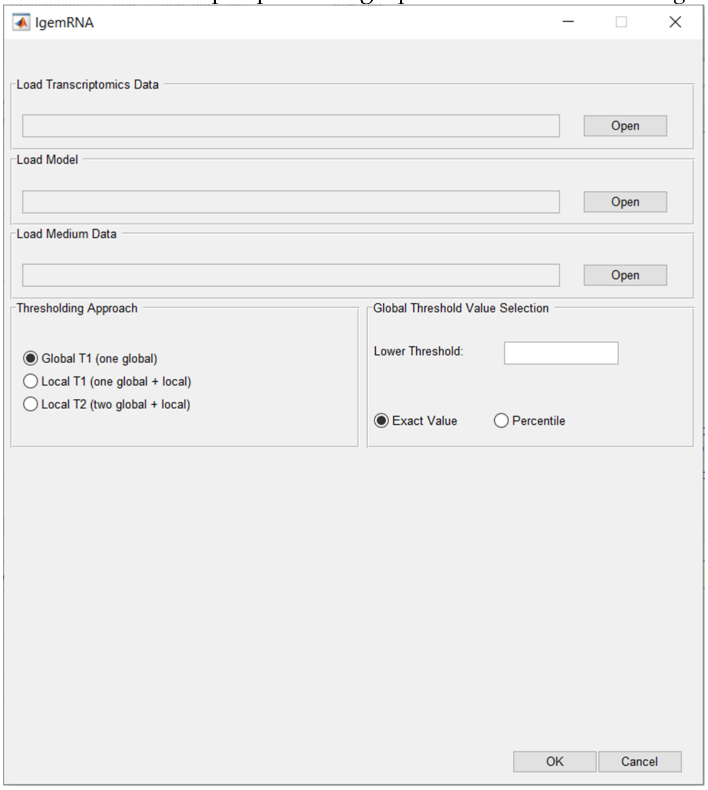

Figure S2. IgemRNA start window.

3. File upload

To access all options in the *IgemRNA* form, the user must supply input data files Figure S3A section. This can be done by pressing the 'Open' button in the corresponding file row and finding the necessary files via File Explorer. Transcriptomics data is required to run non-optimization tasks (Figure S3F) but an additional model file is necessary to access the post-optimization tasks (Figure S3G). Medium composition file is optional if such data is available, the selection of this data file does not extend the form, but specifies the given exchange reaction constraints (upper bounds and lower bounds) on the model for post-optimization tasks analysis. For an organized overview of the analysis and results it is recommended that the necessary data files are located in the Data folder of *IgenRNA* tool (for more details see main publication section Materials and Methods 2.2 Tools functionality description).

Supplementary file S2

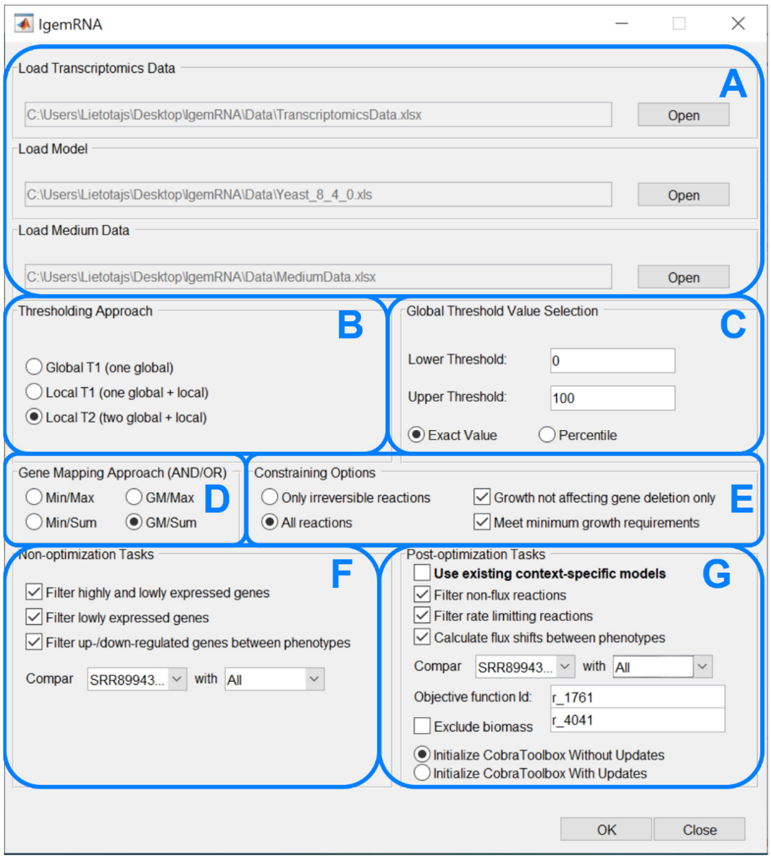

Figure S3. Full *IgemRNA* form

4. Running test cases

In order to perform test cases provided in this user manual, simply run the provided test case scripts via MATLAB environment having initialized CobraToolbox 3.0 beforehand. Test case script file names are given at the end of each test case section.

5. Non-optimization tasks

Non-optimization tasks include several transcriptomics data analysis tasks: filter highly and lowly expressed genes, filter lowly expressed genes, filter up/down regulated genes between different phenotypes or data sets. The results for each task are stored in a different folder within the *Results non-optimization* folder: *Gene expression level comparison, Highly-lowly expressed genes, Lowly expressed genes* (Figure S4).

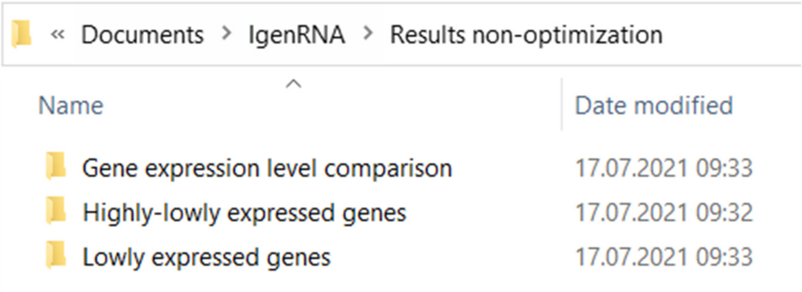

Figure S4. Non-optimization results folder

Supplementary file S2

5.1. Filter highly and lowly expressed genes

Non-optimization task *Filter highly and lowly expressed genes* generates result excel files for each provided transcriptomics data set. File names are assigned based on the provided dataset and phenotype name (from transcriptomics data), the selected thresholding approach (GT1, LT1, LT2) and provided global thresholds values (Figure S5).

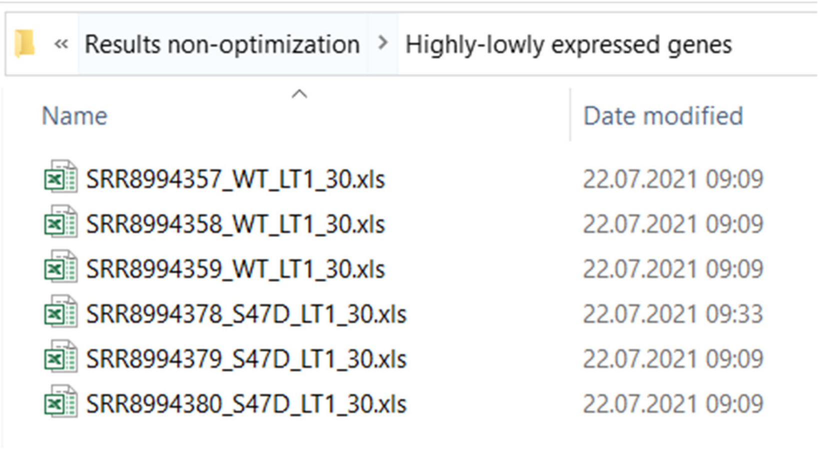

Figure S5. Highly-lowly expressed genes folder

Each excel file contains one sheet with the list of genes provided by transcriptomics data and 4 columns: *GeneId*, *Data* (the expression value), *ExpressionLevel* and *ThApplied*. The *ExpressionLevel* column contains the expression levels determined based on the chosen thresholding approach, provided global and for thresholding approaches (LT1 and LT2) calculated local thresholds. Column *ThApplied* displays whether a local or a global threshold for a specific gene was applied (Figure S6).

	A	B	C	D
1	GeneId	Data	ExpressionLevel	ThApplied
2201	Q0275	0	Low	Global
2202	tM(CAU)Q2	0	Low	Global
2203	Q0285	0	Low	Global
2204	Q0297	0	Low	Global
2205	YDL245C	14	High	Local
2206	YDL242W	48	High	Local
2207	YDL241W	13	High	Local
2208	YDL237W	11	High	Local
2209	YDL236W	14	High	Local
2210	YDL234C	13	High	Local
2211	YDL231C	13	High	Local
2212	YDL230W	24	High	Local
2213	YDL229W	5	High	Local
2214	YDL228C	40140	High	Global
2215	YDL227C	20	High	Local
2216	YDL225W	11	High	Local

Figure S6. Filter highly and lowly expressed genes result file (thresholding approach

LT1) To perform this test case run files from the *Scripts* folder of *IgemRNA* tool:

Supplementary file S2

TestCase_findHighlyLowlyExpressedGenesGT1.m
TestCase_findHighlyLowlyExpressedGenesLT1.m
TestCase_findHighlyLowlyExpressedGenesLT2.m

Or run full tests from the root folder of *IgemRNA*:
ShortTest.m
LongTest.m

5.2. Filter lowly expressed genes

Non-optimization task *Filter lowly expressed genes* generates separate excel result files for each dataset provided in transcriptomics data file. These result files contain filtered gene lists including genes that are below a given threshold based on the selected thresholding approach. File names include dataset and phenotype name (from transcriptomics data file), thresholding approach (GT1, LT1, LT2) name and provided global threshold values (Figure S7).

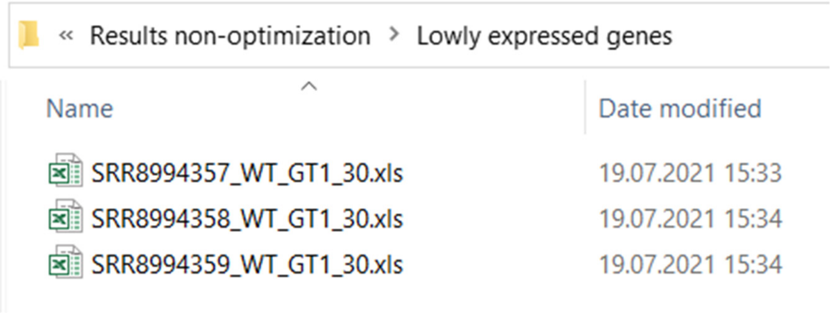

Figure S7. Non-optimization results folder

The result for lowly expressed genes coincides with the provided transcriptomics data format. Each file consists of 4 columns *GeneId*, *Data* (expression value from transcriptomics data), *ExpressionLevel* (Low) and *ThApplied* to show whether a global or local threshold was applied. Only those genes that are below a given threshold (depending on which thresholding approach is applied) are listed in the result files. The test case provided for this task shows genes with expression level below 30 using the Local T2 approach (Figure S8).

Supplementary file S2

	A	B	C	D
1	GeneId	Data	ExpressionLevel	ThApplied
2	YDL248W	0	Low	Global
3	YDL247W-A	0	Low	Global
4	YDL247W	2	Low	Global
5	YDL246C	2	Low	Global
6	YDL245C	6	Low	Local
7	YDL244W	0	Low	Global
8	YDL240C-A	2	Low	Global
9	YDL239C	0	Low	Global
10	YDL238C	1	Low	Global
11	YDL233W	2	Low	Global
12	YDL232W	1	Low	Global
13	YDL224C	0	Low	Global
14	YDL219W	5	Low	Local
15	YDL214C	34	Low	Local
16	tG(GCC)D1	0	Low	Global
17	YDL210W	10	Low	Local

Figure S8. Lowly expressed genes result file

To perform this test case run files from the *Scripts* folder of *IgemRNA* tool:

TestCase_findGenesBelowThresholdGT1.m
TestCase_findGenesBelowThresholdLocal1.m
TestCase_findGenesBelowThresholdLocal2.m

Or run full tests from the root folder of *IgemRNA*:
ShortTest.m
LongTest.m

5.3. Filter up/down regulated genes between phenotypes

Non-optimization task *Filter up/down regulated genes between phenotypes* generates result excel files in the *Gene expression level comparison* folder. Result file names contain dataset and phenotype names for both transcriptomics datasets that have been compared (Figure S9).

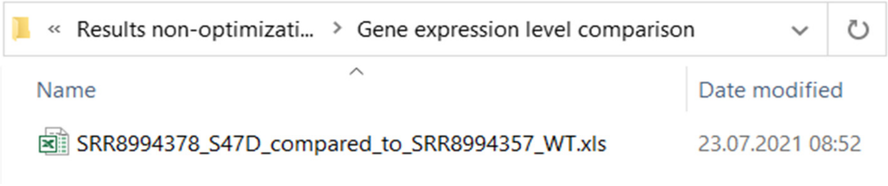

Figure S9. Up/Down regulated genes in comparison to another phenotype

These result excel data files contain a full gene list from the target dataset and the corresponding genes that are found in the source dataset (Figure S10A column). Expression values for both target and source dataset are displayed (Figure S10B, C columns) as well as the determined up/down regulation status (Figure S10D column).

Supplementary file S2

	A	B	C	D
1	GeneId	SRR8994378_S47D_ExpressionValue(target)	SRR8994357_WT_ExpressionValue(source)	Up/Down regulated
2	YDL248W	1	0	up
3	YDL247W-A	0	1	down
4	YDL247W	0	0	equal
5	YDL246C	5	0	up
6	YDL245C	10	14	down
7	YDL244W	6	3	up
8	YDL243C	25	3	up
9	YDL242W	64	48	up
10	YDL241W	20	13	up
11	YDL240C-A	1	1	equal
12	YDL240W	9	0	up
13	YDL239C	0	2	down
14	YDL238C	5	0	up
15	YDL237W	13	11	up
16	YDL236W	10	14	down
17	YDL235C	12	6	up
18	YDL234C	134	13	up

Figure S10. Up/Down regulated genes in comparison to another phenotype result file

To perform this test case run the file *TestCase_findUpDownRegulatedGenes.m* in the *Scripts* folder of *IgemRNA* tool or run full tests from the root folder of *IgemRNA*:

ShortTest.m

LongTest.m

6. Post-optimization tasks

Context-specific models generated by *IgemRNA* post-optimization tasks as well as the results of the analysis performed on these models are saved in the *Results post-optimization* folder of *IgemRNA* tool (Figure S11). The post-optimization tasks are saved in the folders with the corresponding name: Flux-shifts, Non-flux reactions and Rate limiting reactions (for more details see section Materials and Methods 2.2 Tools functionality description).

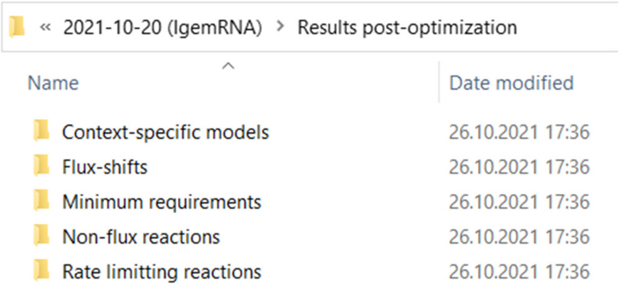

Figure S11. Results folder after post-optimization task execution

To generate context-specific models used for these test cases run the file *TestCase_createContextSpecificModel.m* in the *Scripts* folder of *IgemRNA* tool. Since this script takes a long time to execute, the context-specific model files used for this demonstration have already been provided in the 'Results post-optimization/Context-specific models' folder.

6.1. Filter non-flux reactions

Post-optimization task *filter non-flux reactions* performs an analysis on the created context-specific models of the same phenotype, the name of the phenotype is included in the result file name (Figure S12). This analysis filters those reactions that carry no flux.

Supplementary file S2

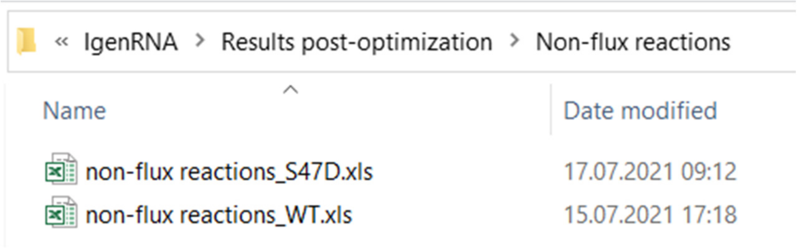

Figure S12. Non-flux reactions result folder

Each result excel file contains a list for each transcriptomics dataset of reactions that carry no flux in the result context-specific model created by integration of the supplied transcriptomics data into the provided model. An additional sheet for all the common non-flux reactions of the same phenotype is also provided in the sheet *Common (phenotype name)* (Figure S13).

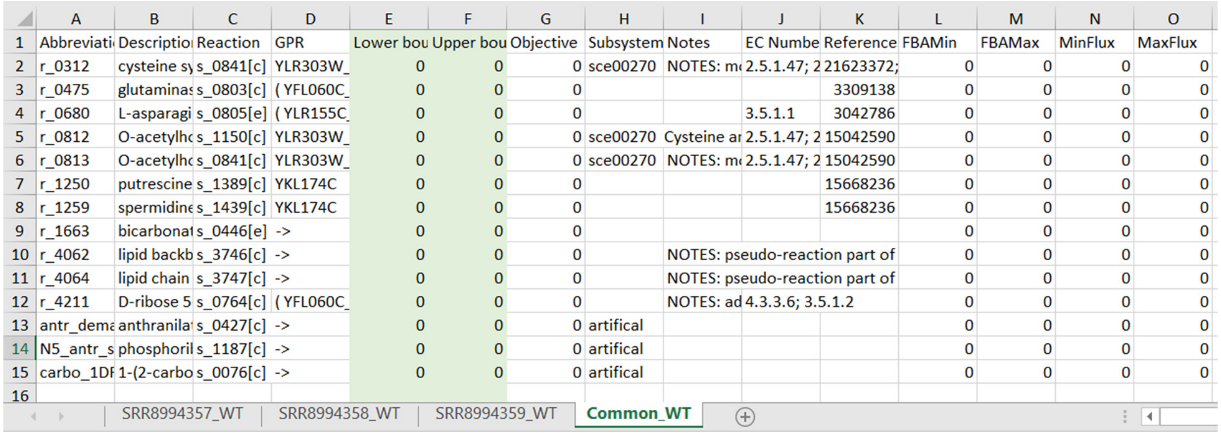

	A	B	C	D	E	F	G	H	I	J	K	L	M	N	O
1	Abbreviati	Descriptio	Reaction	GPR	Lower bou	Upper bou	Objective	Subsystem	Notes	EC Numbe	Reference	FBAMin	FBAMax	MinFlux	MaxFlux
2	r_0312	cysteine sy	s_0841[c]	YLR303W_	0	0	0	sce00270	NOTES: m	2.5.1.47; 2	21623372;	0	0	0	0
3	r_0475	glutaminas	s_0803[c]	(YFL060C_	0	0	0				3309138	0	0	0	0
4	r_0680	L-asparagi	s_0805[e]	(YLR155C_	0	0	0			3.5.1.1	3042786	0	0	0	0
5	r_0812	O-acetylhc	s_1150[c]	YLR303W_	0	0	0	sce00270	Cysteine ar	2.5.1.47; 2	15042590	0	0	0	0
6	r_0813	O-acetylhc	s_0841[c]	YLR303W_	0	0	0	sce00270	NOTES: m	2.5.1.47; 2	15042590	0	0	0	0
7	r_1250	putrescine	s_1389[c]	YKL174C	0	0	0				15668236	0	0	0	0
8	r_1259	spermidine	s_1439[c]	YKL174C	0	0	0				15668236	0	0	0	0
9	r_1663	bicarbonat	s_0446[e]	->	0	0	0					0	0	0	0
10	r_4062	lipid backb	s_3746[c]	->	0	0	0		NOTES: pseudo-reaction part of			0	0	0	0
11	r_4064	lipid chain	s_3747[c]	->	0	0	0		NOTES: pseudo-reaction part of			0	0	0	0
12	r_4211	D-ribose 5	s_0764[c]	(YFL060C_	0	0	0		NOTES: ad	4.3.3.6; 3.5.1.2		0	0	0	0
13	antr_dema	anthranila	s_0427[c]	->	0	0	0	artifical				0	0	0	0
14	N5_antr_s	phosphoril	s_1187[c]	->	0	0	0	artifical				0	0	0	0
15	carbo_1DF	1-(2-carbo	s_0076[c]	->	0	0	0	artifical				0	0	0	0
16															

SRR8994357_WT | SRR8994358_WT | SRR8994359_WT | Common_WT

Figure S13. Wild type non-flux reaction task result

To perform this test case run the file *TestCase_filterNonFluxReactions.m* in the *Scripts* folder of *IgemRNA* tool or run full tests from the root folder of *IgemRNA*:

ShortTest.m

LongTest.m

6.2. *Filter rate limiting reactions*

Post-optimization task *filter rate limiting reactions* performs analysis on the generated context-specific models of the same phenotype, the phenotype name is included in the result files (Figure S14).

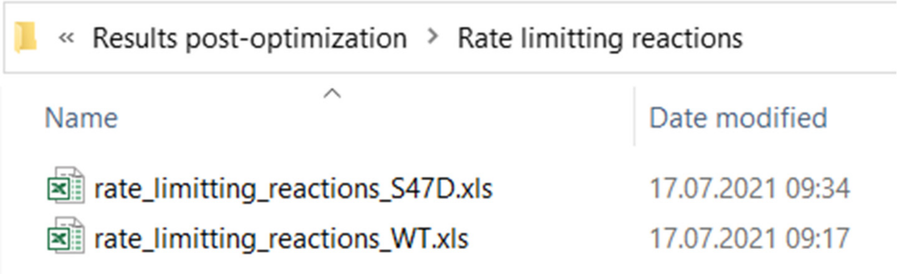

Figure S14. Rate limiting reactions result folder

Supplementary file S2

Each result file contains sheets for each provided transcriptomics dataset of the same phenotype that has been integrated in the supplied model creating context-specific models. An analysis on these context-specific models have been performed in order to filter reactions that have reached the maximal flux (MaxFlux calculated by FVA) based on the upper bound set according to transcriptomics data and GPR associations. An additional sheet for common rate limiting reactions has also been added to the result file where rate limiting reactions that are present in all datasets are listed (Figure S15).

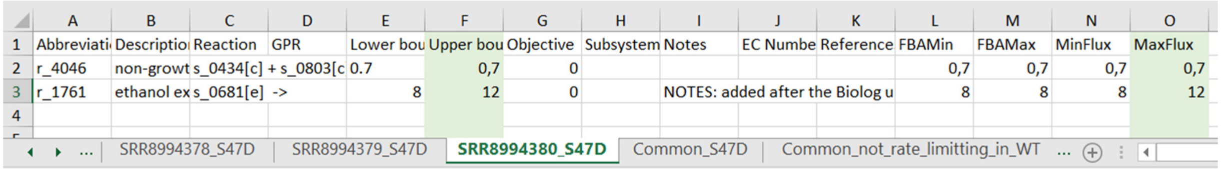

	A	B	C	D	E	F	G	H	I	J	K	L	M	N	O
1	Abbreviati	Descriptio	Reaction	GPR	Lower bou	Upper bou	Objective	Subsystem	Notes	EC Numbe	Reference	FBAMin	FBAMax	MinFlux	MaxFlux
2	r_4046	non-growt	s_0434[c] + s_0803[c		0.7	0,7	0					0,7	0,7	0,7	0,7
3	r_1761	ethanol ex	s_0681[e] ->		8	12	0		NOTES: added after the Biolog u			8	8	8	12
4															

Figure S15. S47D phenotype rate limiting reaction task result

To perform this test case run the file *TestCase_filterRateLimittingReactions.m* in the *Scripts* folder of *IgemRNA* tool or run full tests from the root folder of *IgemRNA*:

ShortTest.m

LongTest.m

6.3. Flux shifts calculation between different phenotypes

Post-optimization task *calculate flux shifts between phenotypes* compares flux values (calculated by FVA on the context-specific models) between two different phenotypes. In this demonstration flux shifts analysis task was performed on the S47D phenotype datasets using Global T1 thresholding approach with the lower global threshold value of 0, phenotype SRR8994358_WT was compared to the wild type dataset SRR8994357_WT of the same thresholding approach and threshold values (Figure S16).

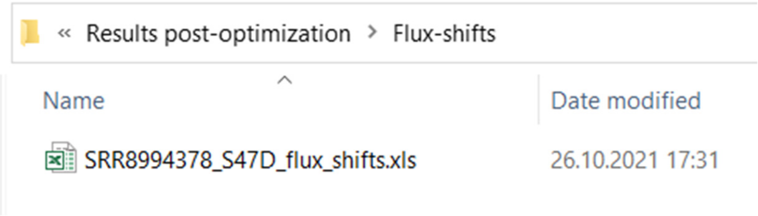

Figure S16. Flux-shifts result folder

Each result file contains a full reaction list that corresponds to the 'Reaction List' sheet in the provided model file as well as additional columns for the calculation results: *MinFlux* and *MaxFlux* values (phenotype that is compared, Figure S17L, M columns), *MinFlux/MaxFlux(dataset name)_(phenotype name)* phenotype that is used for comparison (Figure S17N, O columns) and the *MinFlux/MaxFlux ratio* between these two phenotypes (Figure S17P, Q columns).

Supplementary file S2

	A	B	C	D	E	F	L	M	N	O	P	Q
1	Abbreviatio	Descriptio	Reaction	GPR	LowerBou	UpperBou	MinFlux	MaxFlux	MinFlux_SRR8994357_WT	MaxFlux_SRR8994357_WT	MinFluxRatio	MaxFluxRatio
2	r_0001	(R)-lactate	s_0025[c]	((YDL174	0	51	0	43.2	0	33	1	1,309091
3	r_0002	(R)-lactate	s_0027[m]	((YDL178	0	41	0	41	0	3	1	13,666667
4	r_0003	(R,R)-buta	s_0035[c]	YAL060W	-1000	1000	-8.2	0	-1,200001	0	6,833328	1
5	r_0004	(S)-lactate	s_0063[c]	((YEL039	0	51	0	16	0	5	1	3,2
6	r_0005	1,3-beta-g	s_1543[c]	(YGR032V	0	339	0	0	0	0,245197	1	down
7	r_0006	1,6-beta-g	s_1543[c]	(YGR143V	0	339	0	0	0	0,081925	1	down
8	r_0007	1-(5-phosp	s_0077[c]	YIL020C	0	21	0	8	0	1	1	8
9	r_0012	1-pyrroline	s_0119[m]	YHR037W	-1000	1000	2.269823e	16	0	10	1	1,6
10	r_0013	2,3-diketo-	s_0311[c]	(YEL038V	0	2	0	1	0	1	1	1
11	r_0014	2,5-diamin	s_0142[c]	YOL066C	0	79	0	0	0	0,000328	1	down
12	r_0015	2,5-diamin	s_0141[c]	YBR153W	0	1	0	0	0	0,000328	1	down
13	r_0016	2-aceto-2-	s_0179[m]	((YCL009	0	13	0	7	0	2	1	3,5
14	r_0017	2-amino-4-	s_0148[m]	YNL256W	0	141	0	0	0	0,000021	1	down
15	r_0018	2-aminoad	s_0176[c]	(YER152C	-1000	1000	0	0	0	1,8	1	down

Figure S17. Reaction flux-shifts between two phenotypes

To perform this test case run the file *TestCase_calculateFluxShifts.m* in the *Scripts* folder of *IgemRNA* tool or run full tests from the root folder of *IgemRNA*:
ShortTest.m
LongTest.m.

Most genome scale metabolic models use biomass objective function to simulate biomass accumulation rates, but in many cases such, *S. cerevisiae* Yeast_8_4 version models have a specific wild type function. Optimizing different mutant strain models with deletions and/or specific omics data integration (like transcriptomics data), can yield infeasible optimization solutions although experimental conditions show the opposite. *IgemRNA* has functionality to remove biomass objective function from a model and apply flux distribution with transcriptome and/or medium data sets and analyze results.

7. Nomenclature of file names

IgemRNA also provides standardized output file naming for easier filtering of analysis datasets (Table S1).

Table S1. File name nomenclature.

	Dataset and Phenotype Name	Thresholding Approach	Global Threshold Values
Source	Sheet name in transcriptomics data file (Figure S1B)	Based on the selected thresholding approach in the *IgemRNA* form section B (Figure S3.)	Based on the provided global threshold values in the *IgemRNA* form section C (Figure S3.)
Example/Possible values	SRR8994357_WT	• GT1 (Global T1)<br>• LT1 (Local T1)<br>• LT2 (Local T2)	• 30 (GT1, LT1)<br>• 30_100 (LT2)
